# Small RNA sequencing evaluation of renal microRNA biomarkers in dogs with X-linked hereditary nephropathy

**DOI:** 10.1038/s41598-021-96870-y

**Published:** 2021-08-31

**Authors:** Candice P. Chu, Shiguang Liu, Wenping Song, Ethan Y. Xu, Mary B. Nabity

**Affiliations:** 1 Department of Veterinary Pathobiology, College of Veterinary Medicine & Biomedical Sciences, Texas A&M University, College Station, TX USA; 2 Sanofi, Framingham, MA USA

**Keywords:** Glomerular diseases, Alport syndrome, Transcriptomics, miRNAs, Nephrology, Kidney diseases, Bioinformatics

## Abstract

Dogs with X-linked hereditary nephropathy (XLHN) are an animal model for Alport syndrome in humans and progressive chronic kidney disease (CKD). Using mRNA sequencing (mRNA-seq), we have characterized the gene expression profile affecting the progression of XLHN; however, the microRNA (miRNA, miR) expression remains unknown. With small RNA-seq and quantitative RT-PCR (qRT-PCR), we used 3 small RNA-seq analysis tools (QIAGEN OmicSoft Studio, miRDeep2, and CPSS 2.0) to profile differentially expressed renal miRNAs, top-ranked miRNA target genes, and enriched biological processes and pathways in CKD progression. Twenty-three kidney biopsies were collected from 5 dogs with XLHN and 4 age-matched, unaffected littermates at 3 clinical time points (T1: onset of proteinuria, T2: onset of azotemia, and T3: advanced azotemia). We identified up to 23 differentially expressed miRNAs at each clinical time point. Five miRNAs (miR-21, miR-146b, miR-802, miR-142, miR-147) were consistently upregulated in affected dogs. We identified miR-186 and miR-26b as effective reference miRNAs for qRT-PCR. This study applied small RNA-seq to identify differentially expressed miRNAs that might regulate critical pathways contributing to CKD progression in dogs with XLHN.

## Introduction

Dogs with X-linked hereditary nephropathy (XLHN) have been used as a model of canine chronic kidney disease (CKD) as well as an animal model of human Alport syndrome^1^. In dogs, XLHN causes rapidly progressing juvenile-onset CKD in affected (hemizygous) males and persistent proteinuria in carrier (heterozygous) females^1^. The juvenile-onset CKD in male dogs with XLHN manifests as persistent proteinuria of glomerular origin as early as 3 months of age, followed by progressive azotemia and decreased glomerular filtration rate, developing into end-stage renal failure between 6 months and 1 year of age^1–3^.

Previously, our group used mRNA sequencing (mRNA-seq) to characterize gene expression in dogs with XLHN, comparing rapid and slower progression of disease^3^. In that study, more than 1,947 differentially expressed genes were identified in affected dogs, and TGF-β1 was identified as the top upstream regulator^3^. Although we have described the gene expression related to CKD progression, the driving force for progression is not completely understood.

MicroRNAs (miRNAs, miRs) are small, non-coding RNAs that post-transcriptionally regulate gene expression by binding to the 3ʹ UTR of mRNAs^4–6^. The complex miRNA-mRNA interaction influences various physiological changes and pathological processes^5^. In the kidney, miRNAs play important roles in both kidney development and progression of CKD in humans and animals^7,8^. While highly expressed renal miRNAs have been identified in healthy dogs^9,10^, a comprehensive renal miRNA expression profile in dogs with CKD is lacking. Therefore, we aimed to identify differentially expressed renal miRNAs that may contribute to CKD progression in dogs by regulating gene expression.

In the current study, we used small RNA-seq on RNA isolated from 23 canine kidney biopsies to characterize and compare miRNA expression in 5 dogs with XLHN and 4 controls at 3 clinical time points (T1: onset of proteinuria, T2: onset of azotemia, and T3: advanced azotemia). We compared the performance of 3 different analysis tools: miRDeep2^11^, QIAGEN OmicSoft Studio, and CPSS 2.0^12^. Based on the small RNA-seq data from 23 biopsies, CPSS 2.0 produced better genome mapping rate and identified more differentially expressed miRNAs. We then conducted gene ontology (GO) and pathway analyses to characterize the miRNA targets among sample groups at specific time points. Our results deepen the understanding of the molecular mechanisms in Alport syndrome as well as CKD progression in general. Our findings suggest that differentially expressed miRNAs could serve as diagnostic biomarkers for CKD and potential therapeutic targets for CKD in both dogs and humans.

## Results

### Summary of small RNA-seq method and data

**Figure 1:**
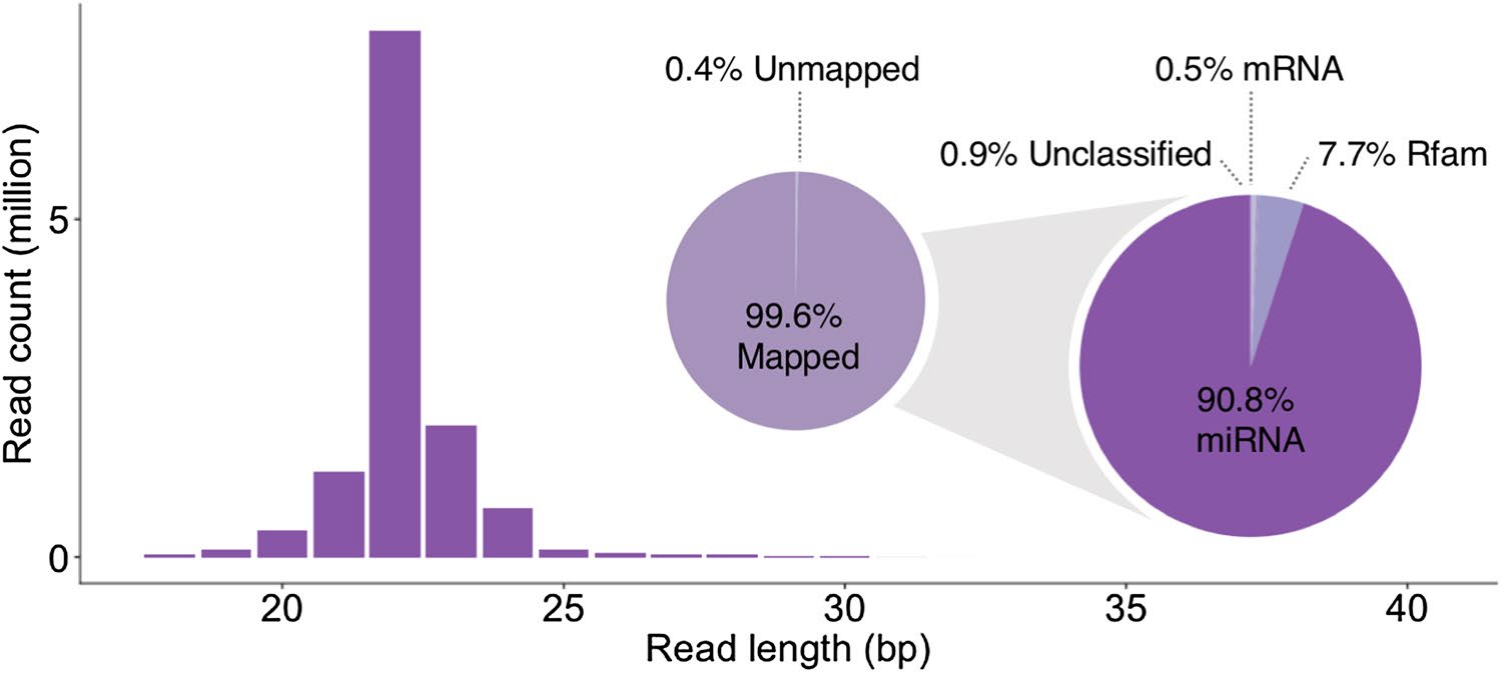
Canine kidney tissue sequencing data with averaged read length distribution (bar chart) and genome mapping results (pie charts). The pie charts indicate almost all reads obtained from 23 kidney tissues mapped to the dog genome. Among the mapped reads, the vast majority (90.8%) belonged to miRNA, and a small population mapped to non-coding RNA (Rfam database; 7.7%) or mRNA (0.5%). The bar chart shows that most sequencing reads were between 21 and 25 nucleotides in length, consistent with miRNAs. The results of CPSS 2.0 were used.

We obtained an average RNA yield of 437.6 ng/µL, with an average 260/280 absorbance ratio of 2.1, from the 23 kidney biopsies collected at 3 clinical timepoints with 3–5 samples in each group (Supplementary Table S1). For each sample, approximately 12 million single-end reads passed the quality control. Overall, 81–99% of reads were mapped to the canine genome (CanFam 3.1). The mean genome mapping rate of CPSS 2.0 (99.6%) was significantly higher than those of OmicSoft Studio (90.0%) and miRDeep2 (88.7%) (Supplementary Fig. S1) (adjusted *P*-value < 0.0001). On average, CPSS 2.0 (167 miRNAs) and OmicSoft Studio (170 miRNAs) detected a significantly higher number of distinct miRNAs than miRDeep2 (124 miRNAs) (adjusted *P*-value < 0.0001). The average miRNA mapping rates were high, ranging from 83 to 94% for each sample (Supplementary Table S1). The representative read length distribution and genome mapping results are illustrated in Fig. 1.

### Principal component analysis (PCA)

**Figure 2:**
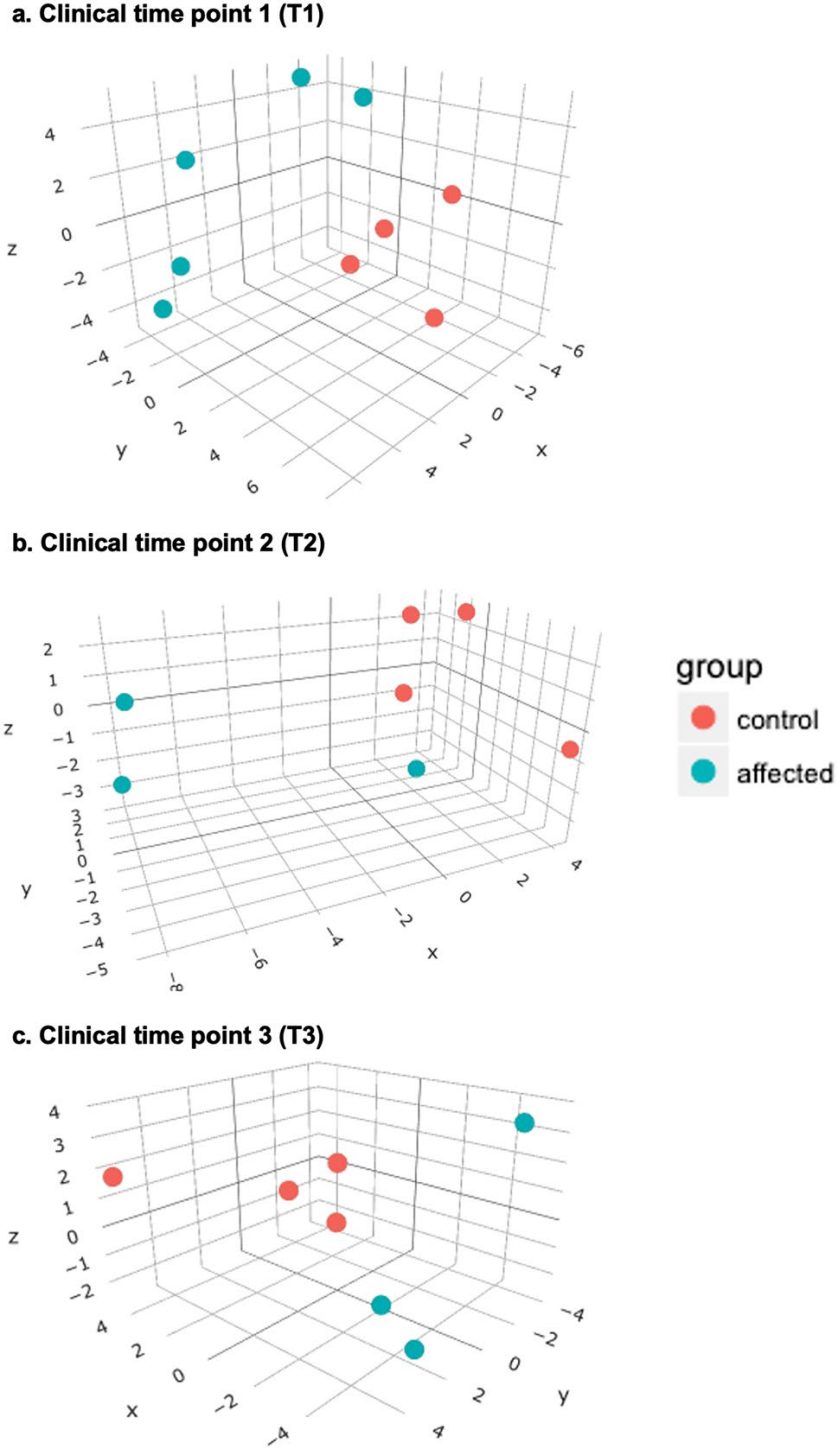
3D principal component analysis (PCA) plots for all canine kidney tissue samples (n = 23), comparing 3–5 affected (XLHN) dogs and 4 controls at each clinical time point (T1, T2, and T3). The x, y, and z axes represent principal components (PC) 1, PC2, and PC3, respectively. The results of CPSS 2.0 were used. (Red: control group; Green: affected group).

PCA was performed to assess the association among samples at each clinical time point (T1, T2, and T3). The PCA of read count tables obtained with all 3 analysis tools showed similar patterns; representative PCA plots for each clinical time point are shown in Fig. 2. Two samples from affected dogs showed a distinctive miRNA expression pattern at T1 but a separation between the affected and control groups was evident on the z-axis (Fig. 2a). At T2, samples were further separated except for 1 affected dog (Fig. 2b). When dogs developed advanced azotemia at T3, a clear separation between affected and control dogs was seen (Fig. 2c).

### Comparison of small RNA-seq analysis tools and differentially expressed miRNAs

**Figure 3:**
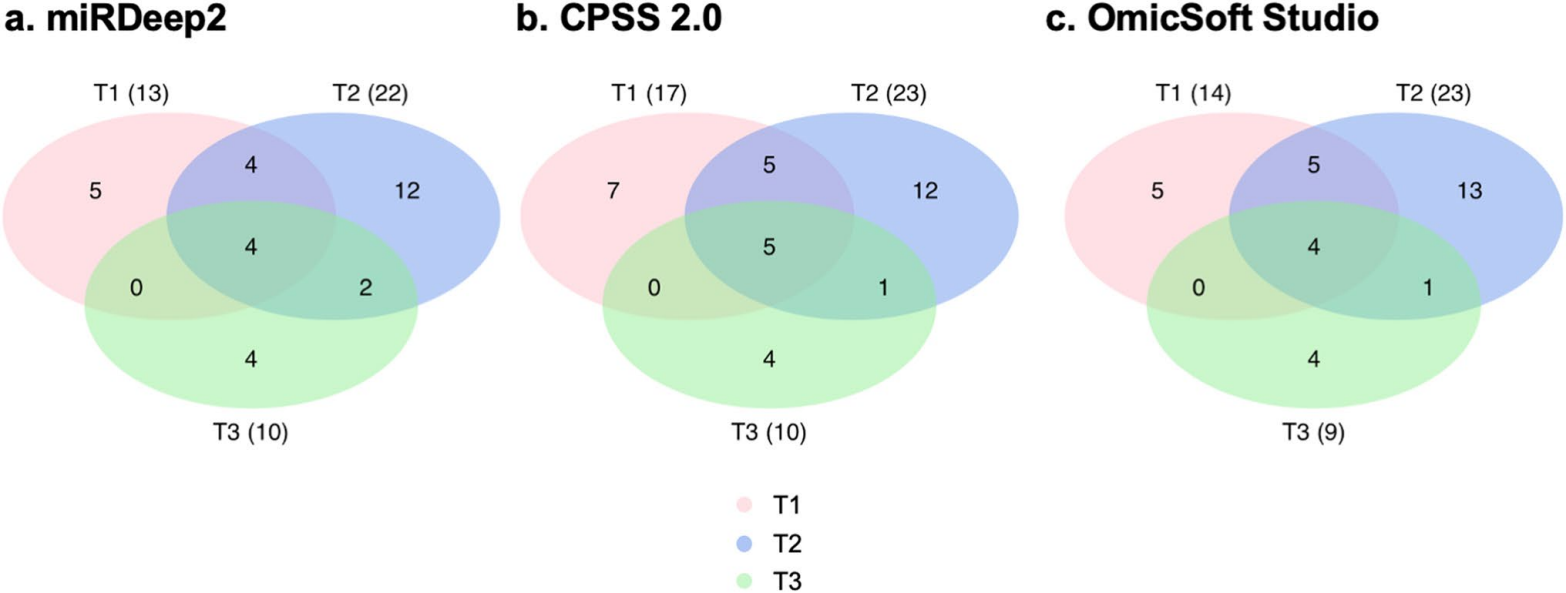
Numbers of differentially expressed miRNAs identified by miRDeep2, CPSS20, and OmicSoft Studio at each clinical time point (T1, T2, and T3). Comparing 3–5 affected (XLHN) dogs with 4 sex- and age-matched controls, up to 23 differentially expressed miRNAs were found at each clinical time point, with the number varying depending on the analysis tool used. The total number of differentially expressed miRs found at each time point for each analysis tool appears in parentheses. Please refer to Table 1 for a complete list of the consistently differentially expressed miRNAs in the center of the Venn Diagrams. Only miRNAs with an adjusted *P*-value < 0.05 and a log twofold change > 1 or < -1 were considered differentially expressed miRNAs. (Pink: T1; Blue: T2; Green: T3).

**Figure 4:**
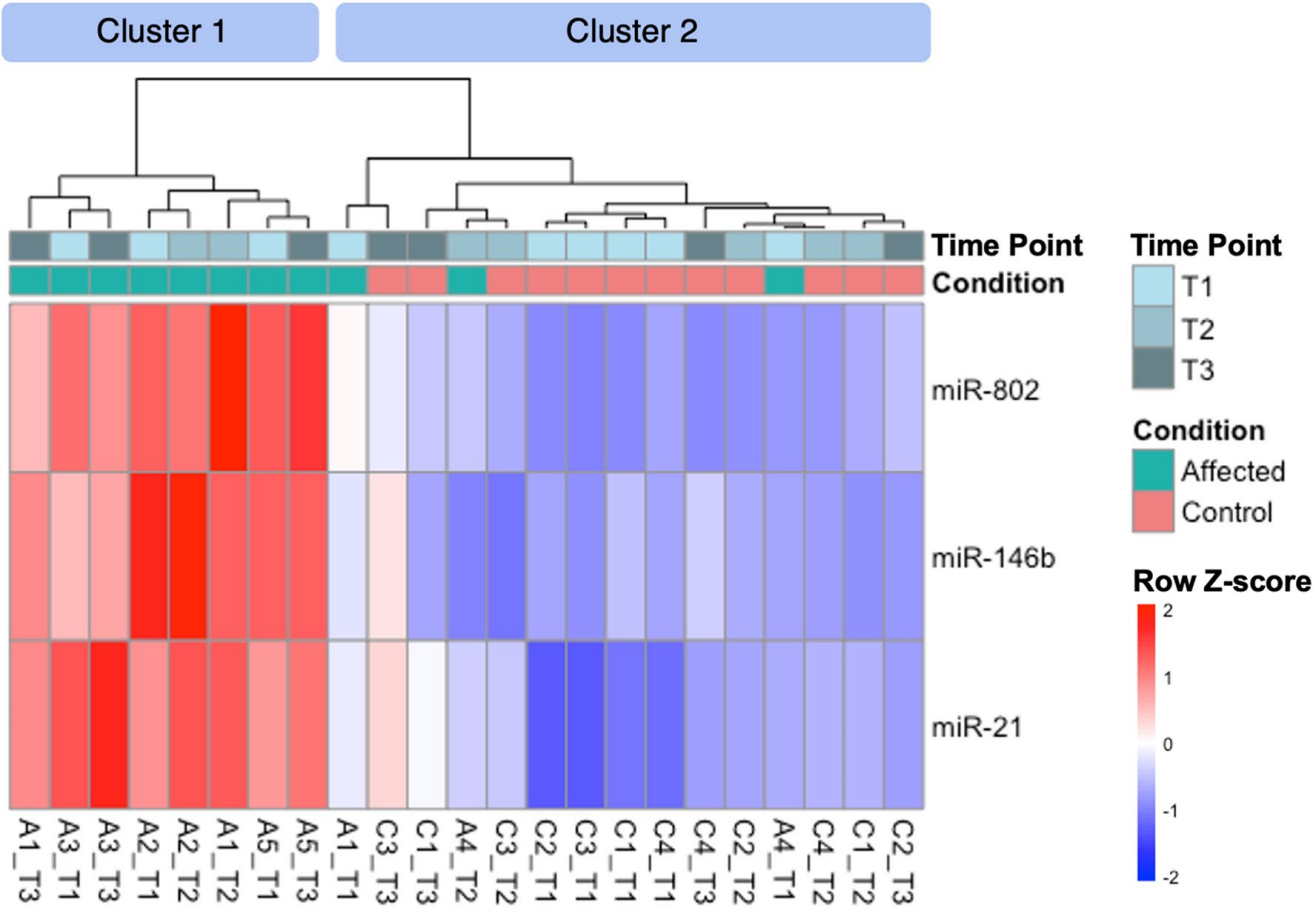
Hierarchical clustering analysis and heatmap of miR-21, miR-146b, and miR-802. Based on sequencing data, miR-21, miR-146b, and miR-802 were upregulated throughout all time points in most XLHN affected dogs (Cluster 1) relative to controls (Cluster 2). (Column names correspond to sample names in Supplementary Table S1: A and C designate affected dogs and controls, respectively; Arabic numerals represent individual dogs in each group; T1, T2, and T3 designate 3 clinical time points). The results from CPSS 2.0 analysis were used.

Overall, up to 23 miRNAs (adjusted *P*-value < 0.05, log2 fold change > 1 or < − 1) were differentially expressed between affected and control dogs at each clinical time point, as shown in the Venn diagrams (Fig. 3). Similar numbers of differentially expressed miRNAs were detected at each time point by all 3 analysis tools. When focusing on miRNAs that were differentially expressed in all 3 time points, miR-21, miR-146b, and miR-802 were consistently upregulated using all 3 analysis tools (Table 1). Hierarchical clustering analysis and heatmap of these miRNAs show that most affected dogs clustered together (Cluster 1 in Fig. 4). The hierarchical clustering results (Fig. 4) agree with the PCA plots in that affected dogs were distinctly separated from the controls for all but 2 dogs (A1 and A4) at T1 (Fig. 2a) and all but 1 dog (A4) at T2 (Fig. 2b).

**Table 1:** Constantly differentially expressed miRNAs throughout all 3 clinical time points using all 3 RNA-seq analysis tools in dogs with X-linked hereditary nephropathy versus controls (T1 = onset of proteinuria, T2 = onset of azotemia, T3 = advanced CKD).

Analysis tools	miRNAs	T1		T2		T3	
		Log2 fold change	Adjusted *P*-value	Log2 fold change	Adjusted *P*-value	Log2 fold change	Adjusted *P*-value
miRDeep2	miR-21	2.33	1.23E−05	1.89	5.31E−04	1.74	1.84E−03
	miR-142	1.65	1.72E−03	2.21	4.42E−04	1.10	4.37E−02
	miR-146b	2.36	8.18E−04	3.31	1.77E−05	2.03	5.06E−04
	miR-802	3.42	1.70E−05	4.37	6.42E−07	2.80	1.54E−04
CPSS 2.0	miR-21	2.30	4.66E−05	1.91	1.25E−03	1.72	3.48E−03
	miR-142	1.67	2.35E−03	2.22	8.25E−04	1.13	4.07E−02
	miR-146b	2.33	1.84E−03	1.85	1.02E−02	2.01	5.47E−04
	miR-147	2.16	2.47E−02	3.27	8.25E−04	2.16	2.47E−02
	miR-802	3.37	6.07E−05	4.20	4.09E−06	2.80	8.03E−05
OmicSoft Studio	miR-21	2.30	5.41E−05	1.95	9.22E−04	1.76	2.93E−03
	miR-146b	2.32	2.32E−03	3.27	8.46E−05	2.06	3.29E−04
	miR-147	2.02	4.69E−02	2.02	4.69E−02	1.94	2.91E−02
	miR-802	3.31	7.33E−05	4.28	2.90E−06	2.86	8.37E−05

### Comparison of differentially expressed miRNAs by qRT-PCR

Meanwhile, miR-142 and miR-147 are detected in only 2 out of 3 analysis tools (Table 1). To further characterize the sequencing results, we performed qRT-PCR for 3 miRNAs (miR-21, miR-142, and miR-147) because miR-21 represents a constantly detected miRNA across all 3 platforms while miR-142, and miR-147 represent miRNAs that were detected in 2 out of 3 platforms. Also, NormFinder analysis was performed on the sequencing data, and 4 miRNAs (miR-186, miR-26b, miR-16, and miR-99a) were identified as promising internal controls. Using qRT-PCR, we examined the expression of these 7 miRNAs using 10 XLHN affected canine kidney tissues sequenced in the current study and 2 canine kidney tissue controls used in a previous study^3^ so there were 2 samples from each group at each time point. The results of the geNorm analysis showed stable expression for 3 of the 4 promising internal controls (miR-186, miR-26b, and miR-16) and indicated that the 2 most stable miRNAs were miR-186 and miR-26b (Supplementary Fig. S2a,b). In the “reference target stability” quality control of qbase +, we further verified the stability of miR-186 and miR-26b and confirmed that they are reliable for normalization using qRT-PCR (Supplementary Fig. S2c).

**Figure 5:**
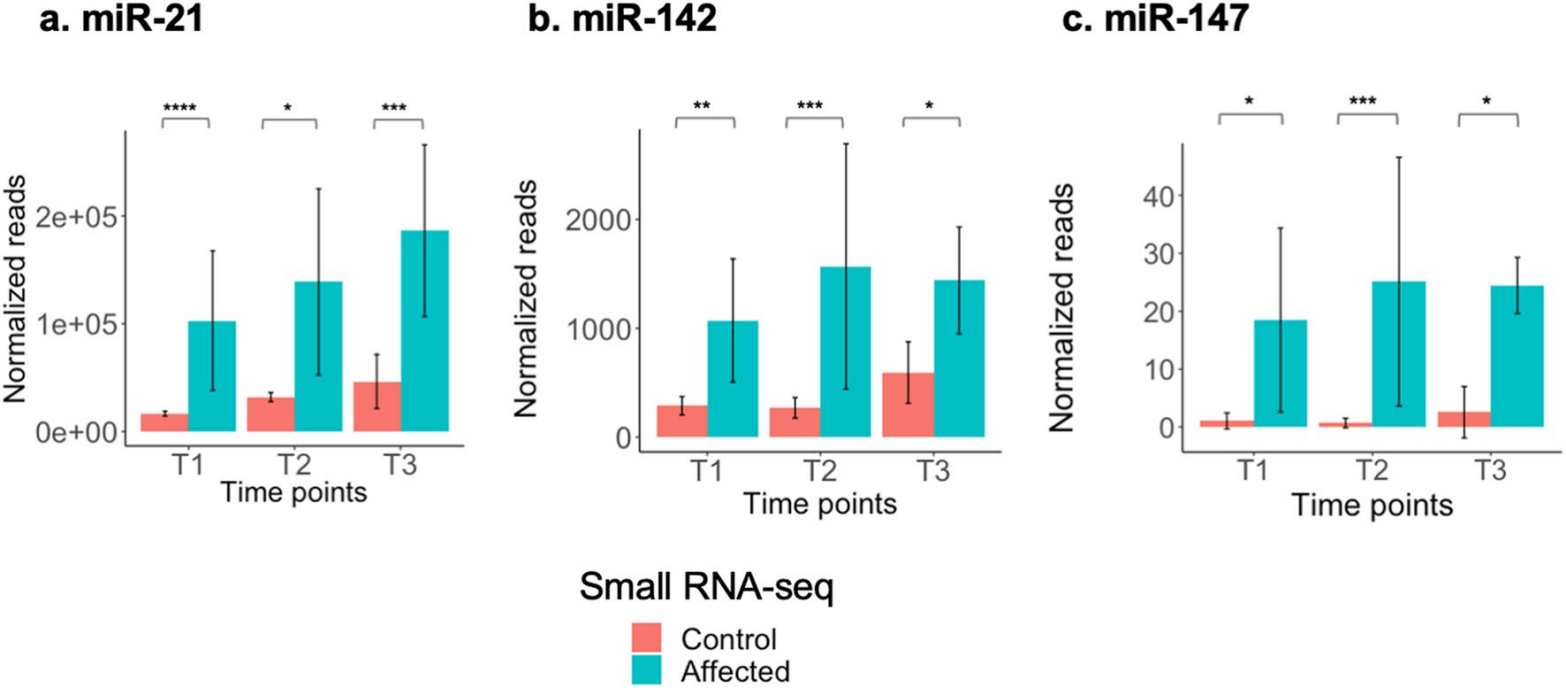
Expression of selected upregulated miRNAs in affected (XLHN) dogs and controls detected by small RNA-seq at each clinical time point (T1, T2, and T3). Expression of (**a**) miR-21, (**b**) miR-142, (**c**) miR-147 are shown. Data are presented as mean value ± standard deviation. The results from CPSS 2.0 analysis were used. (*: *P*-value < 0.05; **: *P*-value < 0.01; ***: *P*-value < 0.001; ****: *P*-value < 0.0001).

The upregulation of miR-21, miR-142, and miR-147 in the kidney tissues of affected dogs at T2 and T3 was detected by small RNA-seq using CPSS 2.0 (Fig. 5) as well as using qRT-PCR (Supplementary Fig. S3). The expression of miR-21, miR-142, and miR-147 based on qRT-PCR showed upward trending as kidney disease progressed in the affected dogs whereas expression in the control dogs was similar at all time points (Supplementary Fig. S3). The qRT-PCR results, along with the higher genome mapping rate, prompted us to use the data generated by CPSS2.0 for the remaining analysis.

### Target prediction, gene ontology (GO), and pathway analysis for differentially expressed miRNAs

The differentially expressed miRNAs identified using CPSS2.0 are listed in Table 2 (adjusted *P*-value < 0.05, log2 fold change > 1 or < − 1). Among them, the top 10 differentially expressed miRNAs were selected as inputs for miRDB^13,14^. To better characterize the changes in target genes, upregulated and downregulated differentially expressed miRNAs were used as 2 separate inputs. In miRDB, only putative target genes with target scores ≥ 90 were included (Supplementary Table S2) and considered satisfactory for the subsequent overrepresentation test in the PANTHER gene ontology (GO) and pathway analyses^15,16^.

**Table 2:** Top differentially expressed miRNAs identified in dogs with X-linked hereditary nephropathy versus controls at 3 time points (T1 = onset of proteinuria, T2 = onset of azotemia, T3 = advanced CKD).

Time point	Regulation	miRNAs	Log2 fold change	Adjusted *P*-value
T1	Up	miR-802	3.37	6.07E−05
		miR-146b	2.33	1.84E−03
		miR-21	2.30	4.66E−05
		miR-147*	2.16	2.47E−02
		miR-150	1.91	1.14E−02
		miR-590	1.69	1.12E−02
		miR-142*	1.67	2.35E−03
		miR-101	1.47	1.76E−02
		miR-31	1.43	2.39E−02
		miR-19a	1.38	4.32E−02
		miR-29a	1.20	7.68E−04
		miR-340	1.14	3.80E−04
	Down	miR-184	− 2.42	1.05E−02
		miR-105b	− 1.70	4.26E−02
		miR-8890	− 1.64	1.84E−03
		miR-196a	− 1.43	3.89E−02
		miR-1301	− 1.30	2.35E−02
T2	Up	miR-802	4.20	4.09E−06
		miR-147*	3.27	8.25E−04
		miR-146b	3.19	1.08E−04
		miR-183	3.03	1.08E−04
		miR-150	2.66	3.06E−04
		miR-182	2.32	8.25E−04
		miR-370	2.25	3.37E−02
		miR-142*	2.22	8.25E−04
		miR-889	2.06	1.59E−03
		miR-96	2.06	2.10E−02
		miR-21	1.91	1.25E−03
		miR-146a	1.85	1.02E−02
		miR-380	1.84	9.17E−03
		miR-410	1.74	9.37E−03
		miR-155	1.60	3.70E−02
		miR-335	1.47	3.37E−02
		miR-381	1.32	3.64E−02
		miR-31	1.32	9.70E−03
		miR-451	1.20	4.63E−02
		miR-29a	1.19	3.35E−03
	Down	miR-184	− 2.56	8.25E−04
		miR-215	− 1.19	1.84E−02
		miR-196a	− 1.09	9.65E−03
T3	Up	miR-802	2.80	8.03E−05
		miR-146b	2.16	5.47E−04
		miR-147*	2.01	2.62E−02
		miR-18a	1.74	1.30E−02
		miR-21	1.72	3.48E−03
		miR-155	1.53	3.23E−02
		miR-34a	1.53	3.23E−02
		miR-142*	1.13	4.07E−02
		miR-708	1.06	4.07E−02
	Down	miR-486	− 1.27	2.62E−02

All listed miRs have a log2 fold change > 1 or < − 1 and adjusted *P*-value < 0.05 in the CPSS 2.0 analysis.

*miR-142 and miR-147 are differentially expressed miRNAs identified at all 3 time points in the CPSS 2.0 analysis, but not in the OmicSoft Studio and miRDeep2 analyses, respectively.

Because of the low number of downregulated miRNAs (27 to 128) and the consequently insufficient miRNA targets, no gene ontology (GO) and pathway analyses results were obtained for downregulated miRNAs at any time point. In contrast, 319 to 729 putative targets of upregulated miRNAs were mapped in PANTHER, identifying 20 unique biological process GO terms, 3 unique Reactome pathways, and 12 unique PANTHER pathways (Supplementary Table S3). “Cellular process” and “RNA metabolic process” and subfamilies of “MAPK cascade” and “regulation of transcription from RNA polymerase II promoter” were the main GO terms enriched in the current study. Specifically, “regulation of transcription from RNA polymerase II promoter” was upregulated at T1 and T3, and “intracellular signal transduction” and “regulation of phosphate metabolic process” were upregulated at T2 and T3.

For Reactome pathway analysis, the “signal transduction” pathway was enriched in affected dogs throughout the progression of kidney disease, and its subfamily pathway the “signaling by TGF-β receptor complex” pathway was also enriched approximately fivefold at T1. The PANTHER pathways, such as the gonadotropin-releasing hormone receptor pathway and Wnt signaling pathway, were enriched in affected dogs at T1 and T2. Ten other enriched PANTHER pathways were identified at T2, but no PANTHER pathway was identified at T3, presumptively because there were fewer miRNA targets (Supplementary Table S3).

## Discussion

Dogs with XLHN have been studied as an example of canine CKD and used as an animal model for human Alport syndrome. The gene expression in XLHN dogs has been partially characterized using qRT-PCR^17^ and microarrays^18^. Previously, our group used mRNA-seq to investigate the gene expression linked to rapid CKD progression in dogs with XLHN^3^. In the current study, we performed small RNA-seq on 23 renal biopsies collected serially from 5 affected dogs and 4 healthy littermates to characterize miRNA expression during CKD progression. The majority of differentially expressed miRNAs in the kidney tissue of affected animals were upregulated, as in previous studies using CKD mouse models^19,20^. We identified up to 23 miRNAs that were differentially expressed at specific clinical time points including 5 miRNAs (miR-21, miR-146b, miR-802, miR-142, miR-147) that were consistently upregulated at all 3 time points (Table 2). Meanwhile, we compared 3 miRNA analysis tools and identified promising miRNA internal controls (miR-186 and miR-26b) for qRT-PCR normalization using canine kidney tissue.

Several studies have documented increased renal miR-21 in different models of CKD, including, but not limited to, Alport syndrome mouse model^21^, B6.MRLc1 mouse model^19^, type 2 diabetes mouse model^22^, and IgA nephropathy patients^23^. MiR-21 is regulated by TGF-β1/Smad3^24–26^ and contributes to renal fibrosis by silencing metabolic pathways^27^. In the current study, we used qRT-PCR to examine the upregulation of renal miR-21 at T2 and T3 in affected dogs. We found that the expression of renal miR-21 did not display an upward trending at the onset of azotemia compared with more advanced azotemia. This is similar to our previous finding in a larger group of XLHN dogs where, based on qRT-PCR, the expression of renal miR-21 did not increase significantly until affected dogs became azotemic^28^. Our results also corroborate our previous finding that TGF-β1 is the top upstream regulator of CKD progression in XLHN dogs^3^.

MiR-146b also has been previously described in the context of CKD. MiR-146b was one of the upregulated miRNAs in the kidney tissue of 12-month-old mice with spontaneous CKD (B6.MRLc1) compared with healthy controls (C57BL/6)^19^. The upregulation of renal miR-146b has also been associated with 4 additional kidney conditions in mouse models (folic acid-induced kidney injury, unilateral ureteral obstruction, bilateral renal ischemia/reperfusion, and cisplatin‐induced kidney injury) with peak expression associated with fibrosis^20^. In the current study, miR-146b reached peak expression at T2, when XLHN dogs became azotemic and had visible fibrotic change on histopathological evaluation^3^. In dogs, mice, and humans, miR-146b and miR-146a are highly homologous miRNAs that differ by only 2 nucleotides. Therefore, they share similar mRNA targets and biological functions^19^. Upregulation of miR-146a has been seen in the kidney tissues of IgA nephropathy patients^29^, a CKD mouse model^19^, a diabetic nephropathy rat model^30^, and human glomeruli in lupus nephritis^31^ and membranoproliferative glomerulonephritis^32^. In the current study, both miR-146a and miR-146b showed an upward trending at T2 in dogs with XLHN, indicating that the function of miR-146a/b is worth further investigation.

Other miRNAs identified in our study, including miR-802, miR-142, and miR-147, have been only rarely described in the context of CKD. In our study, the expression of renal miRNA-802 was upregulated at all 3 time points in the dogs with XLHN. This increased expression of miR-802 has been observed in the kidneys, specifically in the cortical collecting ducts, of mice exposed to high-potassium diets^33^. In vitro, miR-802 targets caveolin-1 (CAV1), decreasing caveolin-1 expression, which in turn increases the surface expression of the renal outer medullary potassium channel and facilitates potassium excretion^33^. In dogs with XLHN, we found CAV1 gene expression increased at T2 and T3^3^, although we would expect the putative target for miR-802 to be downregulated. More studies on canine XLHN miR-802 expression are needed to resolve this discrepancy.

Renal miR-142 (hsa-miR-142-3p) is upregulated in human patients with acute rejection of a renal allograft^34^. Another study suggests the upregulation of miR-142 in renal allografts is due to an influx of lymphoid cells with acute T-cell mediated rejection^35^. Previously, we have demonstrated that T cells are the predominant lymphoid cells infiltrating the kidneys of dogs with XLHN^3^, which could explain the upward trending of miR-142 in the current study. Upregulation of renal miR-147 was detected by RNA-seq in a 3-chloro-1, 2-propanediol (3-MCPD)-induced acute kidney injury mouse model, but its significance has not been discussed^36^. Overall, research on miR-147 and its role in the development of CKD is lacking. To our best knowledge, this study is the first to document upregulation of miR-147 in CKD, which reemphasizes the importance of an unbiased approach using sequencing technology for miRNA discovery^37^.

Using GO terms and pathway analyses, we identified “MAPK cascade” and “regulation of transcription from RNA polymerase II promoter” as the enriched subfamilies of biological processes. Both of these were enriched in patients with CKD and other diseases in previous studies^38,39^. The Reactome pathway analysis supported involvement of the “signal transduction” pathway, particularly the “signaling by TGF-β receptor complex,” consistent with our previous finding that TGF-β1 is the top upstream regulator of CKD progression in XLHN dogs^3^. Additionally, based on PANTHER pathway analysis, several pathways identified (e.g., “gonadotropin-releasing hormone receptor” and “Wnt signaling”) were also related to signal transduction. Indeed, these pathways contained several genes that were downregulated in dogs with XLHN in our previous mRNA-seq study (e.g., ACTG2, ADCYAP1R1, and CACNA1C at T1; MAP3K3, FZD4, ACVR1B, PER1, GATA2, MYH15, and GATA4 at T2)^3^.

Although several tools are available for analysis of RNA sequences, there is a lack of comparisons among these tools. We therefore used 3 analysis tools (CPSS 2.0, OmicSoft Studio, and miRDeep2) and the same version of the canine genome and miRNA annotations with the default settings. Among the 3 analysis tools, miRDeep2 is most widely used for miRNA identification; however, both CPSS 2.0 and OmicSoft Studio surprisingly detected more miRNAs than miRDeep2. With our dataset, the use of CPSS 2.0 resulted in a significantly higher genome mapping rate than that of OmicSoft Studio and miRDeep2. There are several possible reasons for the differences in detected differentially expressed miRNAs among these tools. OmicSoft Studio uses OSA^40^ as the alignment algorithm while both CPSS 2.0 and miRDeep2 use Bowtie^41^. Despite CPSS 2.0 and miRDeep2 employing the same algorithm, Bowtie is operated under different default settings in these 2 programs^11^. The difference can affect the genome mapping rate and the number of miRNAs detected, as we observed. In our small RNA-seq data, CPSS 2.0 has a significantly higher genome mapping rate compared to miRDeep2 and OmicSoft Studio (Supplementary Fig. S1). And, CPSS 2.0 detected similar numbers of miRNAs as OmicSoft Studio but outperformed miRDeep2 (Supplementary Fig. S1). Also, at each time point, CPSS 2.0 identified more or equal numbers of differentially expressed miRNAs compared to OmicSoft Studio and miRDeep2 (Fig. 3). In our hands, CPSS 2.0 appears to be the preferred analysis tool for small RNA-seq analysis. However, further evaluation is needed as the conclusion cannot be made using a single dataset.

In all 3 analysis tools, miR-21 was repeatedly detected at all time points, whereas both miR-142 and miR-147 were detected by only 2 of the 3 analysis tools. Therefore, we incorporated qRT-PCR to detect the expression of miR-21, miR-142, and miR-147 (Supplementary Fig. S3). The qPCR results support the miR-142 and miR-147 expression detected by CPSS 2.0 but additional samples are needed for verification. In general, PCR is often used to verify sequencing results. It has been argued that qRT-PCR suffered from GC-bias^42^ and inconsistency among different assays^43^. Also, the expression level of a particular miRNA depends on the normalization method used. Although small nuclear RNA (snRNA) (e.g., U6 snRNA) are frequently used for qRT-PCR normalization, snRNAs differ structurally and functionally from miRNAs. The difference in nucleic acid composition, length, and secondary structure could potentially introduce variation between a snRNA control and miRNAs of interest^44^. In order to identify miRNAs for use in normalization, we first applied NormFinder^45^ to the small RNA-seq data and then selected 4 miRNAs that appeared to be stably expressed (miR-186, miR-26b, miR-16, and miR-99a) for further assessment. Based on our qRT-PCR results, miR-186, miR-26b, and miR-16 had similarly low M values in the geNorm analysis (Supplementary Fig. S2), and all of these have been proposed as internal controls in previous renal studies^10,46^. In particular, miR-186 was identified as one of the most stably and ubiquitously expressed miRNAs across 16 types of canine tissues, including kidney^10^. Also, miR-26b has been described as a suitable internal control for glomerular miRNA quantification in IgA nephropathy patients^46^. Our data support miR-186 and miR-26b are more suitable for reference miRNAs for future studies in canine kidneys.

One limitation of the current study was the inability to perform microscopic evaluation and RNA isolation on the same kidney biopsy. If we were able to evaluate the sample used for small RNA-seq by light microscopy, we might have found that the unexpected similarity in renal miRNA expression patterns between a few of the affected dogs at T1 and T2 and the controls (Fig. 2) could be explained by having sampled an area of the kidney that was less affected by the disease than expected, for example. Another limitation of our study is the implementation of in silico analysis and the lack of verification of the miRNA targets and performance of functional studies. To minimize false positive results, we have chosen the most credible tool, miRDB^13,14^, and adapted the most stringent rule of a target score higher than 90, out of the range of 50–100. If samples were available, the study could potentially benefit from in situ hybridization to confirm the downregulation of predicted miRNA targets. Lastly, the miRNA expression profile used whole kidney cortex and therefore mostly represents the tubulointerstitium. Future studies could use laser capture microdissection or single-cell RNA-seq to help narrow down the cell types of interest to achieve a more specific miRNA expression.

In the current study, we applied 3 analysis tools (QIAGEN OmicSoft Studio, miRDeep2, and CPSS 2.0) to identify differentially expressed miRNAs in dogs with CKD using small RNA-seq and qRT-PCR. Based on our data, CPSS 2.0 seems to have advantages over the other two tools in its high genome mapping rate and the comprehensive identification of differentially expressed miRNAs. We identified 5 miRNAs (miR-21, miR-146b, miR-802, miR-142, miR-147) that were consistently upregulated in dogs with CKD compared to age-match controls. The upregulation of miR-21 and miR-146b correspond to the onset of azotemia and could be associated with fibrosis, whereas miR-802, miR-142, and miR-147 were novel miRNAs that warrant further investigation in the context of CKD. The putative targets of these miRNAs and the enriched pathways aligned well with our mRNA-seq study^3^ and further characterize the development and the progression of CKD that involves the signal transduction and TGF-β receptor complex. In both studies, we acquired biopsies at defined clinical stages of disease in the same dogs, which was helpful to accurately compare miRNA expression between controls and dogs with XLHN throughout disease progression. MiRNAs found in the current study may be diagnostic biomarkers for CKD and potential therapeutic targets for CKD in both dogs and humans.

## Methods

### Animals

The dogs evaluated in this study were part of a colony with XLHN maintained at Texas A&M University^2^. XLHN is caused by a 10-base deletion in the gene encoding the ⍺5 chain of type IV collagen. The affected males develop juvenile-onset CKD that progresses to end-stage renal disease, as previously described^2^. Overall, 9 dogs were included in the study. At each clinical time point, 3–5 affected dogs and 4 age-matched unaffected littermates were included (Supplementary Table S1). All dogs were raised according to standardized protocols, and no treatments were given to these dogs. All animal experiments were performed in accordance with the relevant guidelines and regulations set forth by the Texas A&M University Institutional Animal Care and Use Committee represented by the approved study protocol #2010-132 and #2005-129. The study was carried out in compliance with the ARRIVE guidelines (http://www.nc3rs.org.uk/page.asp?id=1357, last accessed on May 24, 2021).

### Clinical phenotypes of XLHN dogs

Clinical progression in the 5 affected dogs was determined by serial monitoring of serum and urine biomarkers of kidney disease, which allowed comparison of dogs at standardized clinical time points^47^: T1 (n = 5; onset of proteinuria: defined as the presence of microalbuminuria for 2 consecutive weeks [E.R.D. HealthScreen Canine Urine Test strips, Loveland, CO, USA]); T2 (n = 3; onset of azotemia: serum creatinine ≥ 1.2 mg/dL); and T3 (n = 3; serum creatinine ≥ 2.4 mg/dL). On average, the affected dogs reached the first clinical time point (T1) at 13.2 weeks of age (range: 11–17 weeks), the second clinical time point (T2) at 23.3 weeks of age (range: 21–26 weeks), and the last clinical time point (T3) at 29.6 weeks of age (range: 24–33 weeks) (Supplementary Table S1).

### RNA isolation and sequencing

All samples (n = 23) were homogenized in RLT Buffer (Qiagen, Valencia, CA) using a Bead Ruptor Mill Homogenizer (Omni International, Kennesaw, GA). Total RNA, including small and miRNA, was isolated using the MirVana miRNA Isolation Kit (ThermoFisher Scientific, Waltham, MA) following the manufacturer’s protocol. Total RNA concentration and 260/280 absorbance ratio were determined by the NanoDrop 2000 (Thermo Fisher Scientific, Wilmington, DE, USA) (Supplementary Table S1). The samples were processed using the TruSeq Small RNA Library Preparation Kit (Illumina, San Diego, CA, USA) according to the manufacturer’s protocol. The following steps were included in small RNA library preparation: small RNA filtration on PAGE gel, adapter ligation, reverse transcription, PCR product purification, and library quality testing by the Agilent 2100 Bioanalyzer (Agilent Technologies, Santa Clara, CA, USA) and the ABI StepOnePlus Real-Time PCR System. Samples were then sequenced using the Illumina HiSeq 4000 (50 bp single-end) at the sequencing facility (BGI America, Cambridge, MA, USA) to reach the expected output of 10 million reads per sample.

### Small RNA-seq data analysis

Preprocessing of small RNA-seq reads and quality control were performed by the sequencing facility (BGI). Low quality reads were defined as reads with more than 4 bases with a quality score less than 10 or reads with more than 6 bases with a quality score less than 13. Briefly, low quality reads, adapter contaminants (no insert reads and 5’ primer contaminants) and reads less than 18nt were filtered out. Length distribution was inspected to confirm the composition of the small RNAs as shown in Fig. 1. Data analysis was carried out using 3 analysis tools: (1) QIAGEN OmicSoft Studio (Version 10.0.1.48), a commercial next-generation sequencing and omics data analysis platform, (2) miRDeep2^11^ (version 2.0.0.8, Latest Update: May 2016), a popular algorithm for miRNA identification, and (3) CPSS 2.0 (http://114.214.166.79/cpss2.0/index.html), a web-based small RNA-seq analysis tool^12^. For all 3 analyses, the canine genome (CamFam 3.1) and miRBase (release 21) were used with default settings. MiRDeep2 was performed using the High Performance Research Computing resources provided by Texas A&M University (http://hprc.tamu.edu) in the Linux operating system (version 2.6.32). For all 3 tools, un-normalized raw read counts were used to perform differential expression and statistical analysis with the identical script using DESeq2^48^ (release 3.3) in R (version 3.6.2) as previously described^3^. The 3D PCA plots were made using Plotly (https://plotly.com/r/). We used the Benjamini–Hochberg procedure^49^ to adjust *P*-values for multiple testing. An adjusted *P*-value < 0.05 was set for the selection of differentially expressed miRNAs.

### Target prediction, GO terms, and pathway analysis

For differentially expressed miRNAs at each time point, we selected the top 10 upregulated (fold change > 2) and down-regulated (fold change < 2) miRNAs as input for miRDB^13,14^. miRDB is an online database for miRNA target prediction that hosts predictive miRNA targets in 5 species: humans, mice, rats, dogs, and chickens. There are 453 miRNAs targeting 170,435 genes in the database (http://mirdb.org/, last accessed on Dec 30, 2020). All miRNA targets are assigned a target score from 50 to 100 determined by the MirTarget algorithm^50^. We used the target score of 90 as a cutoff value since higher scores represent more statistical confidence. The list of miRNA targets was passed on to gene ontology (GO) and PANTHER pathway analysis using the *Canis familiaris* reference list (20,141 genes) and the Overrepresentation Test (released on December 5, 2017) in PANTHER^15^ version 13.1 with the default setting (Protein ANalysis THrough Evolutionary Relationships, http://www.pantherdb.org/, released on February 3, 2018). Also, manually curated Reactome pathway analysis^16^ version 58 (released on December 7, 2016) was used. The updated PANTHER Overrepresentation Test incorporates Fisher’s Exact Test with false discovery rate multiple test correction, and adjusted *P*-value < 0.05 was set as the cutoff value.

### qRT-PCR

Small RNA-seq data generated by the 3 analysis tools were analyzed using NormFinder^45^ to identify suitable miRNAs as candidates for internal controls in qRT-PCR. Samples remaining after small RNA-seq (n = 10) were used for qRT-PCR. Additionally, control samples used in a previous study^3^ (n = 2) were recruited. Overall, there were 2 samples from the affected group and 2 from the control group at each time point, for a total of 12 samples. The miRCURY LNA miRNA PCR Assays (Qiagen, Germany) were used for the miRNA targets: miR-21 (Cat. no. YP00204230), miR-142 (Cat. no. YP02102101), and miR-147 (Cat. no. YP00204368). The miRCURY LNA miRNA PCR Assays (Qiagen, Germany) were used for the miRNA internal controls: miR-186 (Cat. no. YP00206053), miR-26b (Cat. no. YP00205953), miR-16 (Cat. no. YP00205702), and miR-99a (Cat. no. YP00204521). First, total RNA isolated from kidney biopsies was diluted to the concentration of 10 ng/μL. Next, the miRCURY LNA RT Kit (Qiagen, Germany) was used in a 10 μL reaction for RT consisting of 2 μl of 5X Reaction Buffer, 5 μL RNase-free water, 1 μL enzyme mix (omitted for no reverse transcriptase control wells), and 2 μL diluted RNA (10 ng/μl). The RT reaction was performed using a T100 Thermal Cycler (Bio-Rad, UK) with a protocol of 60 min at 42 °C (reverse transcription), 5 min at 95 °C (inactivation), followed by storage at -20 °C. Although the cDNA is stable for up to 5 weeks, all PCRs were performed within 18 days from the RT reaction. For PCR, the miRCURY LNA SYBR Green PCR Kit (Qiagen, Germany) was used to make a 10 μL reaction consisting of 5 μL SYBR Green Master Mix, 1 μL PCR Primer Mix, 1 μL RNase-free water, and 3 μL 1:15 diluted cDNA (omitted for no template control wells). Mater Mix and cDNA were distributed in Hard-Shell 384-Well Standard PCR Plates (Bio-Rad, UK) in duplicates by the epMotion 5075 Automated Liquid Handling System (Eppendorf, Germany). PCR was performed using a CFX384 Touch Real-Time PCR Detection System (Bio-Rad, UK) with a protocol of 2 min at 95 °C (initial heat activation), 40 cycles of 10 s at 95 °C (denaturation) and 60 s at 56 °C (annealing), followed by a melting curve analysis at 60–95 °C. Negative controls included RNase-free water only, no reverse transcriptase (NRT), and no template controls (NTC) to ensure no genomic DNA contamination was present.

For the internal controls, the Cq values of the candidate miRNAs were further analyzed by geNorm^51^ in the qbase + software^52^. Candidate miRNAs for internal controls were ranked by geNorm^51^ based on stability (M value) and coefficient of variation. The algorithm of geNorm^51^ then calculates the normalization factor (V value) and determines the optimal number of internal controls. Lastly, the candidate miRNAs were analyzed for “reference target stability” quality control in qbase +^52^. Target miRNAs were normalized based on the 2^−ΔΔCq^ method^53^, using miR-186 and miR-26b as reference miRNAs.

## Supplementary Information

Supplementary Figure S1.

Supplementary Figure S2.

Supplementary Figure S3.

Supplementary Table S1.

Supplementary Table S2.

Supplementary Table S3.

## Data Availability

Raw sequencing reads generated from this study are deposited at the NCBI sequence read archive (SRA) under BioProject ID PRJNA664365.
