# Supplementary material for: Small RNA sequencing evaluation of renal microRNA biomarkers in dogs with X-linked hereditary nephropathy
Source: Sci Rep. 2021 Aug 31;11:17437. doi: 10.1038/s41598-021-96870-y (PMC8408228; doi:10.1038/s41598-021-96870-y)
Supplement: Supplementary file 1 — Supplementary Figure S1. [file 41598_2021_96870_MOESM1_ESM.docx]

**Small RNA sequencing evaluation of renal microRNA biomarkers in dogs with X-linked hereditary nephropathy**

Candice P. Chu^1^, Shiguang Liu^2^, Wenping Song^2^, Ethan Y. Xu^2^, Mary B. Nabity^1,*^

^1^Department of Veterinary Pathobiology, College of Veterinary Medicine & Biomedical Sciences, Texas A&M University, College Station, TX, USA. ^2^Sanofi, Framingham, MA, USA.

^*^Correspondence and requests for materials should be addressed to MBN. (email: mnabity@cvm.tamu.edu)

| 1. **Genome mapping rate (%)**   **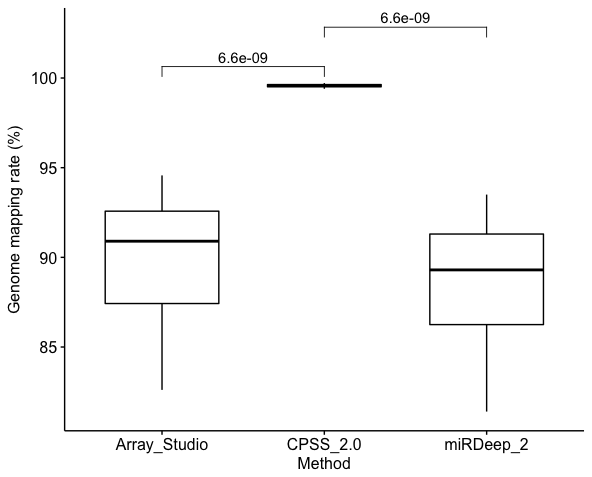**  OmicSoft_Studio | 1. **Number of miRNAs detected**   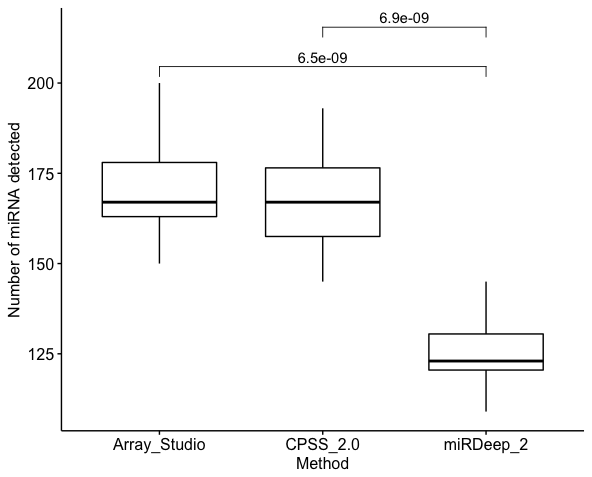  OmicSoft_Studio |  |
| --- | --- | --- |
|  | | |

**Supplementary Figure S1. Boxplots of genome mapping rate (a) and number of miRNA detected (b).** Wilcoxon test was performed in a pair-wise manner with Benjamini-Hochberg Procedure for multiple testing. Adjusted P-values < 0.05 were shown in the plots. CPSS 2.0 had higher mean genome mapping rate than other 2 alignment tools. For number of miRNAs detected, only miRNAs having more than 1 reads per million raw reads were counted. Both OmicSoft Studio and CPSS 2.0 detected higher number of miRNAs than miRDeep 2.
