# Supplementary material for: Small RNA sequencing evaluation of renal microRNA biomarkers in dogs with X-linked hereditary nephropathy
Source: Sci Rep. 2021 Aug 31;11:17437. doi: 10.1038/s41598-021-96870-y (PMC8408228; doi:10.1038/s41598-021-96870-y)
Supplement: Supplementary file 2 — Supplementary Figure S2. [file 41598_2021_96870_MOESM2_ESM.docx]

**Small RNA sequencing evaluation of renal microRNA biomarkers in dogs with X-linked hereditary nephropathy**

Candice P. Chu^1^, Shiguang Liu^2^, Wenping Song^2^, Ethan Y. Xu^2^, Mary B. Nabity^1,*^

^1^Department of Veterinary Pathobiology, College of Veterinary Medicine & Biomedical Sciences, Texas A&M University, College Station, TX, USA. ^2^Sanofi, Framingham, MA, USA.

^*^Correspondence and requests for materials should be addressed to MBN. (email: mnabity@cvm.tamu.edu)

| 1. **geNorm M values (gene expression stability measure) of promising miRNA internal controls**   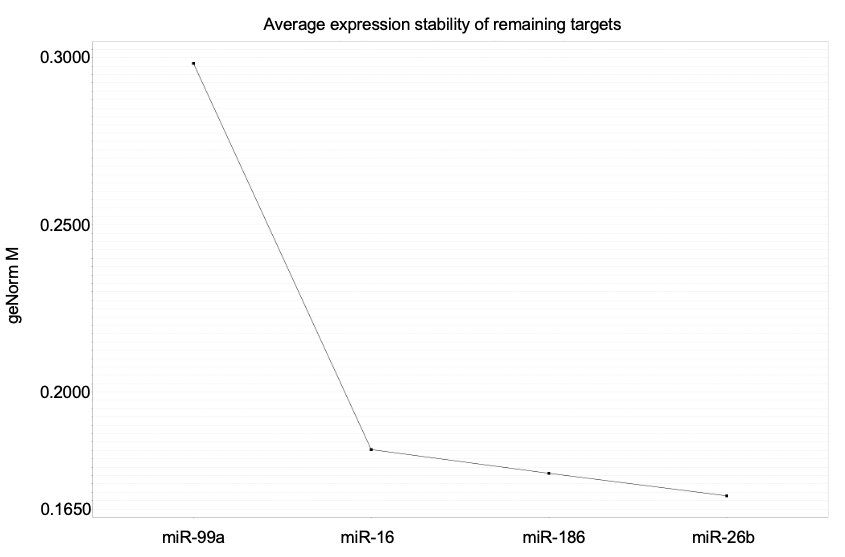 |
| --- |
| 1. **geNorm V values (average pairwise variation) of promising miRNA internal controls**   **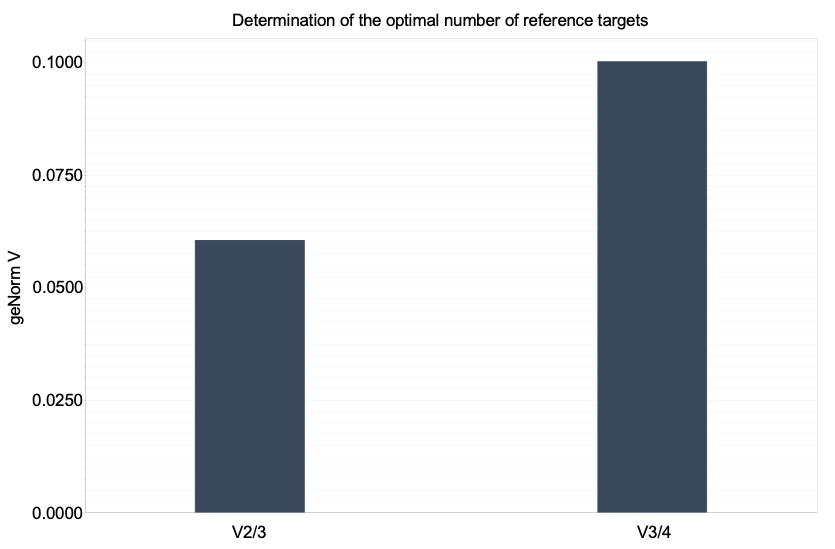** |

1. **Reference target stability**

**
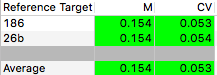
**

**Supplementary Figure S2. geNorm M values (a), V values (b) of promising miRNA internal controls, and the “reference target stability” quality control (c).** The geNorm analysis was initiated using 12 samples and 4 promising miRNAs. (a) The genes are listed from the least stable one on the left to the most stable one on the right. A geNorm M value of less than 2 indicates stable expression of the miRNAs. (b) Based on the geNorm V values (geNorm V < 0.15 when comparing a normalization factor based on the 2 or 3 most stable miRNAs), the optimal number of internal controls is 2. (c) The reference target stability is applied to verify the selected reference genes (miR-186 and miR-26b). The green color indicates the selected reference genes have passed quality control. Therefore, miR-186 and miR-26b were used as internal controls.
