# Supplementary material for: Small RNA sequencing evaluation of renal microRNA biomarkers in dogs with X-linked hereditary nephropathy
Source: Sci Rep. 2021 Aug 31;11:17437. doi: 10.1038/s41598-021-96870-y (PMC8408228; doi:10.1038/s41598-021-96870-y)
Supplement: Supplementary file 3 — Supplementary Figure S3. [file 41598_2021_96870_MOESM3_ESM.docx]

**Small RNA sequencing evaluation of renal microRNA biomarkers in dogs with X-linked hereditary nephropathy**

Candice P. Chu^1^, Shiguang Liu^2^, Wenping Song^2^, Ethan Y. Xu^2^, Mary B. Nabity^1,*^

^1^Department of Veterinary Pathobiology, College of Veterinary Medicine & Biomedical Sciences, Texas A&M University, College Station, TX, USA. ^2^Sanofi, Framingham, MA, USA.

^*^Correspondence and requests for materials should be addressed to MBN. (email: mnabity@cvm.tamu.edu)

| **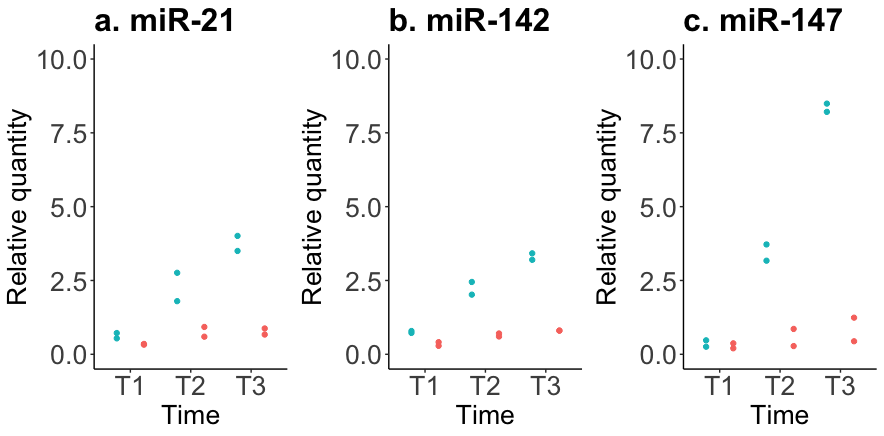** | **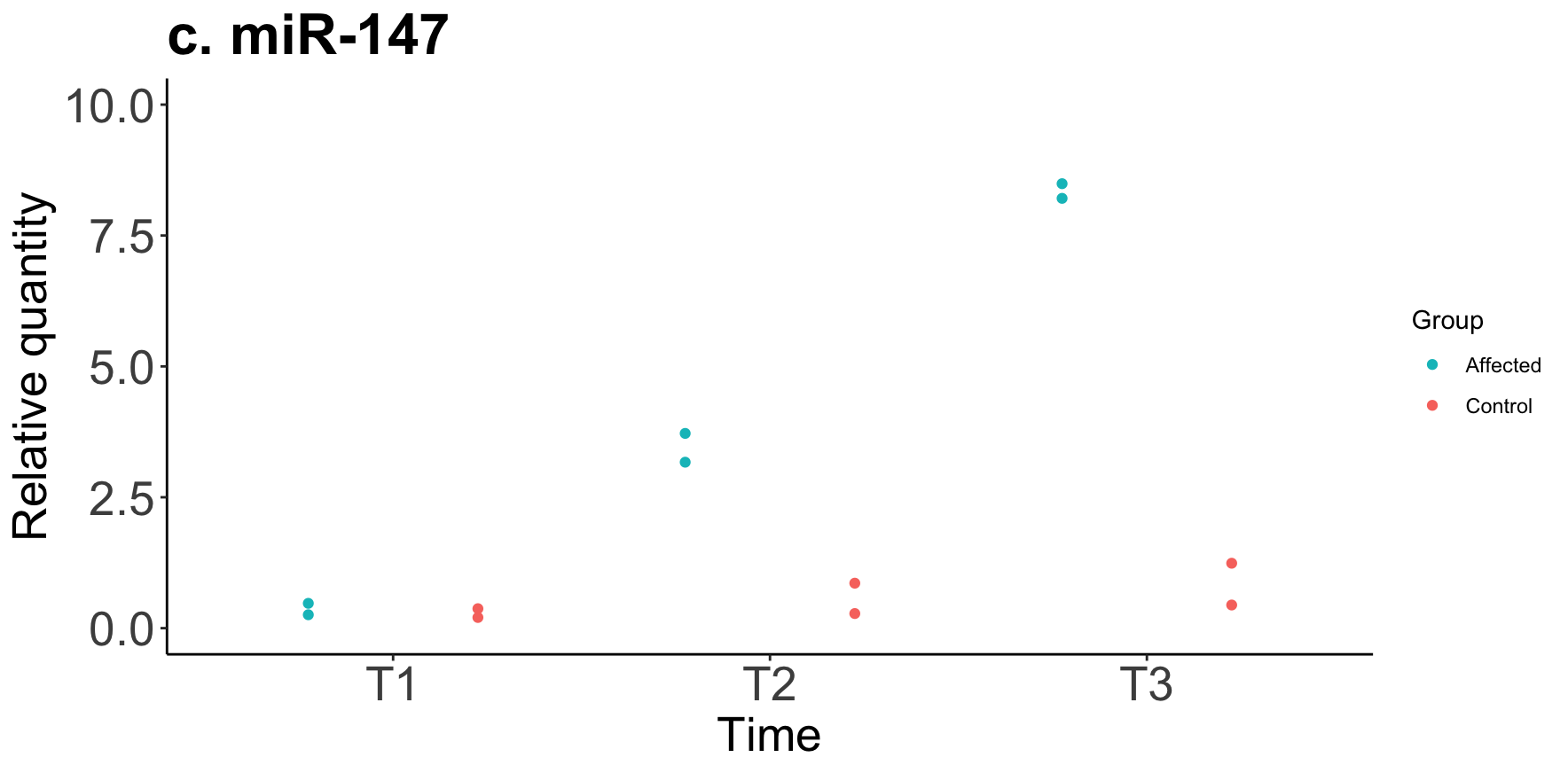** |
| --- | --- |

**Supplementary Figure S3.** **Expression of selected upregulated miRNAs in 2 affected (XLHN) dogs and 2 controls detected by qRT-PCR at each clinical time point (T1, T2, and T3).** Expression of: (a) miR-21, (b) miR-142, and (c) miR-147.
